# *Toxoplasma gondii* and *Neospora caninum* in invasive wild boars (*Sus scrofa*) and hunting dogs from Brazil

**DOI:** 10.1016/j.ijppaw.2024.100951

**Published:** 2024-06-06

**Authors:** Patricia Parreira Perin, Carmen Andrea Arias-Pacheco, Lívia de Oliveira Andrade, Jonathan Silvestre Gomes, Adrian Felipe de Moraes Ferreira, Rafael Oliveira Pavaneli, Fabiana Alves Loureiro, Ana Luíza Franco, Wilson Junior Oliveira, Talita Oliveira Mendonça, Natália de Oliveira Zolla, Mateus de Souza Ribeiro Mioni, Rosangela Zacarias Machado, Luiz Daniel de Barros, João Luis Garcia, Rafaela Maria Boson Jurkevicz, Ana Carolina Cavallieri, Estevam G. Lux Hoppe

**Affiliations:** a Department of Pathology, Reproduction, and One Health, School of Agricultural and Veterinary Studies, São Paulo State University. Prof. Paulo Donato Castellane Road, 14884900, Jaboticabal, São Paulo, Brazil; b Protozoology Laboratory, Department of Preventive Veterinary Medicine, Center of Agrarian Sciences, State University of Londrina. Celso Garcia Cid Road, 86055900, Londrina, Paraná, Brazil

**Keywords:** Foodborne diseases, Hunting, One health, Sarcocystidae, Suidae

## Abstract

The wild boar, an impactful invasive species in Brazil, is subject to population control activities, which often include the use of hunting dogs. Hunters commonly consume wild boar meat, which is also used to feed their dogs, posing a risk of *Toxoplasma gondii* infection for humans and both *T. gondii* and *Neospora caninum* for dogs. The study aimed to investigate the prevalence of infection in wild boars (n = 127) and hunting dogs (n = 73) from São Paulo, Rio Grande do Sul, and Paraná states. We employed histopathological, serological (indirect fluorescent antibody test), and molecular techniques (endpoint polymerase chain reaction). Histopathology slides of wild boar tissue (central nervous system, heart, skeletal muscle, liver, spleen, kidney, gastrointestinal tract, pancreas, lymph nodes, and thyroid) sections revealed no *T. gondii* or *N. caninum* cysts (0/47). Antibodies anti-*T. gondii* were detected in 35/108 (32.4%) and anti-*N. caninum* in 45/108 (41.7%) wild boars. Only 2/18 (11.1%) wild boar tissue homogenate samples tested positive for *T. gondii* on endpoint PCR. Hunting dogs showed antibodies against *T. gondii* in 62/73 (85%) and against *N. caninum* in 31/73 (42%). The presence of antibodies against *T. gondii* and *N. caninum* in wild boars and hunting dogs, along with *T. gondii* DNA detection in wild boars, indicates the circulation of these parasites. Educating hunters on preventing these foodborne diseases, including zoonotic risks, is crucial.

## Highlights

• *Toxoplasma gondii*'s overall prevalence in wild boars from Brazil was 27.6%.
• *Neospora caninum*'s seroprevalence in wild boars from Brazil was 41.7%.
• *T. gondii*'s seroprevalence in hunting dogs from Brazil was 85%.
• *N. caninum*'s seroprevalence in hunting dogs from Brazil was 42%.
• *N. caninum* infection was more frequent in female wild boars.

## Graphical abstract

**Figure:**
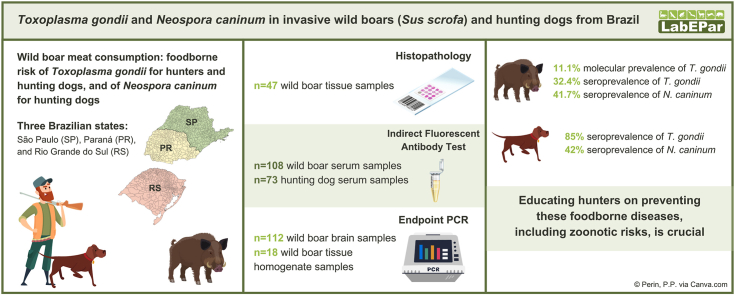

## 1 Introduction

The wild boar (*Sus scrofa* Linnaeus, 1758) has been recognized as one of the 100 most detrimental invasive species globally due to its extensive geographical distribution, escalating conflicts with humans, and challenging management (Lowe et al., 2000). Since its domestication, the species has spread worldwide, establishing wild populations in diverse regions, including Brazil, dating back to the 15th century (Donkin, 1985; Frantz et al., 2016). Verifiable sightings of wild boars are documented in 20 out of the 27 Brazilian states, with a notable surge in population over the past three decades, particularly in the southern, southeastern, and midwestern regions of the country (Hegel et al., 2022). The presence of highly anthropized areas seems conducive to the dispersal of this invasive species (Hegel et al., 2022). The hunting of wild boars was officially sanctioned throughout Brazil by the Brazilian Institute of the Environment and Renewable Natural Resources (IBAMA) in 2013, recognizing the wild boar as a harmful species in all its variations, lineages, breeds, and degrees of crossbreeding with domestic pigs (IBAMA, 2013). The utilization of hunting dogs for wild boar population control activities has been permitted in Brazil since 2019 (IBAMA, 2019).

Wild boars are omnivores and consume a diverse of plant and animal matter (Schley and Roper, 2003), making them suitable indicators for monitoring environmental contamination (Bártová et al., 2006; Reiterová et al., 2016). Additionally, the species scavenges, exposing itself to an increased risk of infection (Lizana et al., 2021). Wild boars serve as potential reservoirs of various zoonotic and animal-specific parasites and are often related to the interface between wild and rural areas (Villa et al., 2023), increasing the chance of pathogen spillover.

Hunting dogs may face increased exposure to infections compared to other canine populations, such as household dogs, due to their proximity to forest remnants, rural areas, and wild animals (Machačová et al., 2016).

*Neospora caninum* and *Toxoplasma gondii* were initially considered the same organism until 1988 (Machačová et al., 2016). Morphologically similar, these obligate intracellular cyst-forming protozoa (Apicomplexa: Sarcocystidae) have a broad host spectrum and closely interface with both domestic and wild cycles (Donahoe et al., 2015; Dubey, 2021).

Wild and domestic canids serve as definitive hosts for *N. caninum* (Machačová et al., 2016), and cattle is considered the main intermediate host, although there is increasing evidence that the intermediate host range is wider than anticipated (Fereig and Nishikawa, 2020). Human infection has not been confirmed, so *N. caninum* is not considered a zoonotic parasite (Calero-Bernal et al., 2019). This parasite is the causative agent of neosporosis, one of the major causes of abortion in cattle, with substantial economic losses to the dairy and beef industries (Goodswen et al., 2013).

Wild and domestic felids serve as the definitive hosts for *T. gondii*, with potentially any homeothermic species, including humans, acting as intermediate hosts (Dubey, 2021). *Toxoplasma gondii* oocysts exhibit remarkable resistance and can remain infectious for over a year under various environmental conditions (Almería and Dubey, 2021). Approximately one-third of the global human population is estimated to be chronically infected with *T. gondii*, with clinical manifestations being more severe in immunocompromised individuals, neonates, and pregnant women. South America, notably, exhibits the highest prevalence of latent toxoplasmosis among pregnant women (Rostami et al., 2021).

In Brazil, hunters, their families, and friends commonly consume meat from wild boars they have hunted (Machado et al., 2021). This meat is also used to supplement the diets of hunting dogs and is sometimes distributed locally, despite being against current laws. When consumed undercooked, this meat poses a risk of *T. gondii* infection for humans (Choi et al., 1997). Additionally, poor hygiene practices during handling and culinary preparation can lead to cross-contamination, increasing the risk of human infection (Jones and Dubey, 2012). Providing raw and undercooked offal and meat from wild boars to reward hunting dogs represents a potential route of infection for both parasites (Machačová et al., 2016).

Therefore, the prevalence of infection by *N. caninum* and *T. gondii* was investigated in wild boars from three Brazilian states (São Paulo, Rio Grande do Sul, and Paraná). The seroprevalence of *N. caninum* and *T. gondii* was also accessed in hunting dogs from two Brazilian states (São Paulo, Rio Grande do Sul, and Paraná).

## 2 Materials and methods

### 2.1 Ethics statement

All procedures adopted in this study were in accordance with international standards. The present study was approved by the Animal Ethics Committee (CEUA) of the School of Agricultural and Veterinary Studies (FCAV), São Paulo State University (Unesp), Jaboticabal, SP, Brazil (protocol numbers 1190/19, 3683/20, 4141/20 and 3413/21) and the Chico Mendes Institute for Biodiversity Conservation (ICMBio) (SISBio protocol numbers 62641–2, 67,577–1, 84,726–1 and 80,862–2).

### 2.2 Study area

**Fig. 1:**
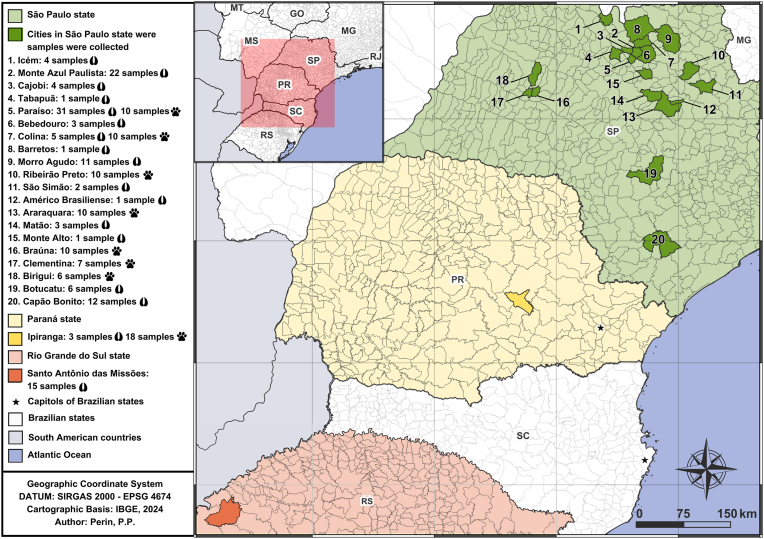
Map highlighting the cities where wild boar and hunting dog samples were obtained, Brazil, 2024.

The wild boar samples were collected in agricultural properties within highly anthropized areas in eighteen municipalities from three Brazilian states: Monte Azul, Morro Agudo, Barretos, Cajobi, Matão, São Simão, Paraíso, Colina, Bebedouro, Monte Alto, Tapabuã, Paraíso, Icém, Capão Bonito, Botucatu, and Américo Brasiliense (São Paulo), Ipiranga (Paraná), and Santo Antônio das Missões (Rio Grande do Sul). The samples from hunting dogs were collected in eight municipalities from Two Brazilian states: Paraíso, Colina, Araraquara, Bonfim Paulista, Birigui, Braúna and Clementina (São Paulo), and Ipiranga (Paraná). Details on the vegetation, climate, and major economic activities of the study areas were described by Oliveira et al. (2023). The number of wild boar and dog samples collected by location can be found in Fig. 1.

### 2.3 Sample acquisition

#### 2.3.1 Wild boar samples

Wild boars were sampled from April 2019 to July 2023. Information about estimated weight, sex, pregnancy status, date of sampling, and place of capture was registered. Regarding the age group, the animals were categorized as juveniles (less than 6 months old), or adults (more than 6 months old) (Oliveira et al., 2023). The sampling strategy carried out without biostatistical criteria due to the lack of data on the wild boar population, depended on the hunting success of our partner hunters, resulting in a convenience sampling approach. The number of obtained animals, sex, and age profiles were influenced by chance and the hunt success rate.

Blood samples were collected by cardiac puncture, using disposable syringes and sterile 18 gauge needles. They were stored in plain vacuum collection tubes, centrifuged (2000×*g* for 10 min), and the obtained sera were stored at −20 °C until serological analysis. Brain samples were collected in DNAse/RNAse-free microtubes and stored at −20 °C for molecular analysis. Brain, heart, tongue, and diaphragm fragments were placed in sterile bags, stored in isothermal boxes with ice, homogenized as described previously (Machado et al., 2021), and stored at −20 °C for molecular analysis. The central nervous system, heart, skeletal muscle, liver, spleen, kidney, gastrointestinal tract, pancreas, lymph nodes, and thyroid fragments were stored in 10% buffered formaldehyde solution (pH 7,4) for 24–48 h. After fixation the fragments were embedded in paraffin, 3 μm thick sections were adhered to glass slides, and stained with hematoxylin-eosin for histopathological analysis. All the samples were processed and stored in the Parasitic Diseases Laboratory (LabEPar, FCAV, Unesp).

#### 2.3.2 Hunting dog samples

Blood samples were obtained by venipuncture from hunting dogs from October 2021 to January 2023. They were stored in plain vacuum collection tubes, centrifuged (2000×*g* for 10 min) at the laboratory (LabEPar, FCAV, Unesp), and the obtained sera were stored at −20 °C until serological analysis. Information about sex and feeding habits (just commercial dry dog food or commercial dry dog food plus human food scraps; wild boar meat consumption-yes or no; if yes, cooked or raw) were registered. The sampling depended on the hunter's willingness to allow their dogs to be sampled for the study, resulting in a convenience sampling approach.

### 2.4 Histopathology

Histological slides were examined under an optical microscope (Olympus BX-51) at various magnification levels to identify *T. gondii* and *N. caninum* cysts and other possible alterations.

### 2.5 Indirect fluorescent antibody test (IFAT)

The presence of IgG antibodies against *T. gondii* and *N. caninum* in serum samples of wild boars and dogs was evaluated by Indirect Fluorescent Antibody Test (IFAT). Commercial slides containing *T. gondii* or *N. caninum* tachyzoites in marked wells were used (IMUNODOT®, Jaboticabal, São Paulo, Brazil). Test serum samples and positive and negative controls were diluted at 1:64 for *T. gondii* and 1:50 for *N. caninum* for wild boar serum, and at 1:40 for *T. gondii* and 1:50 for *N. caninum* for dog serum. The IFAT protocol was conducted as described by Baldini et al. (2022), except for the conjugates: Anti-Pig IgG and Anti-Dog IgG were used for wild boar and dog serum samples, respectively (Sigma-Aldrich, St. Louis, Missouri, EUA). Known positive and negative serum samples were used as positive and negative controls for both species. The slides were observed at 400× magnification under a microscope (Olympus BX-51) equipped with fluorescent light. Test positivity was determined through observation of the total peripheral fluorescence of the tachyzoites (Paré et al., 1995). Seropositive samples from wild boars were two-fold serially diluted to determine the endpoint antibody titer.

### 2.6 Molecular analyses

DNA was extracted from the brain, heart, tongue, and diaphragm homogenate following Bag et al. (2016), and macerated brain fragments with the DNeasy Blood and Tissue® kit, following the manufacturer's protocol (QIAGEN, Hilden, Alemanha). The DNA extraction products were stored at −20 °C until molecular tests. The presence of amplifiable DNA was verified through endpoint Polymerase Chain Reaction (PCR), using the endogenous mammalian gene that encodes glyceraldehyde-3-phosphate dehydrogenase (GAPDH) as described previously (Birkenheuer et al., 2003).

The extraction product of all the homogenate and brain samples were screened for *T. gondii* via endpoint PCR using the TOX4/TOX5 primers (Homan et al., 2000) targeting the noncoding REP-529 genomic DNA fragment of *T. gondii*. The PCR reaction was carried out in a volume of 25 μL containing 2.5 μL of 10X PCR Mg-free buffer, 0.75 μL of 50 mM MgCl_2_, 0.5 μL of 10 mM of dNTP mix, 0.1 μL 2 Platinum™ Taq DNA Polymerase (Thermo Fisher Scientific, Waltham, Massachusetts, EUA), 500 nM of each primer, 2.5 μL of DNA template, and 13.65 μL of nuclease-free water. The PCR cycling conditions on a Nexus thermal cycler (Eppendorf, Hamburg, Germany) were set as initial denaturation at 94 °C for 7 min, followed by 35 cycles of denaturation at 94 °C for 1 min, primer annealing at 60 °C for 1 min, extension at 72 °C for 1 min, and final extension at 72 °C for 10 min. DNA extracted from *T. gondii* RH strain tachyzoites was used as the positive control and ultrapure water was used as the negative control. The amplification products were submitted to electrophoresis in 1% agarose gel, stained with ethidium bromide, and visualized in a Geldoc XR photodocumenter (Bio-Rad®, Hercules, Califórnia, EUA). Positive samples were genotyped using eleven markers (SAG1, 5′ and 3′, SAG2, alt.SAG2, SAG3, BTUB, GRA6, c22-8, c29–2, L358, PK1, and Apico) following the polymerase chain reaction-restriction fragment length polymorphism (PCR-RFLP) protocol of Su et al. (2010).

All brain samples' extraction product was screened for *N. caninum* via endpoint PCR using the Np21/Np6 that anneals to the Nc-5 region (Yamage et al., 1996). The PCR reaction was carried out in a volume of 25 μL containing 2.5 μL of 10X PCR Mg-free buffer, 0.75 μL of 50 mM MgCl_2_, 0.5 μL of 10 mM of dNTP mix, 0.1 μL 2 Platinum™ Taq DNA Polymerase (Thermo Fisher Scientific, Waltham, Massachusetts, EUA), 2.0 μL of each 400 nM of each primer, 2.0 μL of DNA template, and 15.15 μL of nuclease-free water. The PCR cycling conditions on a Nexus thermal cycler (Eppendorf, Hamburg, Germany) were the same as Okeoma et al. (2004) described, except for the primer annealing temperature, which was 62 °C. DNA extracted from *N. caninum* tachyzoites was used as the positive control and ultrapure water was used as the negative control. The amplification products were submitted to electrophoresis in 1% agarose gel, stained with ethidium bromide, and visualized in a Geldoc XR photodocumenter (Bio-Rad®, Hercules, Califórnia, EUA).

### 2.7 Data analysis and statistics

An animal was considered positive if its samples were positive in at least one of the techniques (IFAT or endpoint PCR for wild boars and IFAT for dogs). Prevalence was determined as the number of positive animals divided by sample size (n), and confidence intervals (CI) were calculated with the Wilson test.

For wild boars, the prevalence of infection (*Toxoplasma gondii*, *Neospora caninum*) was compared to the host sex, age group (young and adult), pregnancy status, season of the year, biome (Atlantic rainforest, Pampa grasslands, and Cerrado savanna), mesoregions, and states. For dogs, the prevalence of infection (*T. gondii*, *N. caninum*) was compared to the host sex, feeding habits (only commercial dry dog food, mainly human food scraps, wild boar meat consumption; if yes, cooked, or raw), and mesoregion.

Initially, the association between the outcome variable (prevalence of infection) and the explanatory variables was investigated using univariate analysis (Fisher's exact test). Variables with p-value <0.20 in the univariate analysis were subjected to simple logistic regression analysis. Then, based on the variables with a value of p-value <0.10 in the simple logistic regression analysis, we attempted to obtain multiple models with p-value <0.05 using multiple logistic regression. The wild boars' weight was evaluated with the Shapiro-Wilk test for normality, then compared with the prevalence using the non-parametric Wilcoxon test, the p-value was set at < 0.05. All statistical analyses were performed using the software R version 4.2.1 and Epi Info.

## 3 Results

Blood and tissue samples were collected from 127 wild boars: 61 females (21 pregnant) and 66 males; 30 young and 97 adults; 109 from São Paulo, 3 from Paraná, and 15 from Rio Grande do Sul. All the samples were obtained in highly anthropized agricultural areas across three biomes: Atlantic rainforest, Pampa grasslands, and Cerrado savanna. A summary of results obtained for wild boar samples regarding host characteristics and geographical descriptors can be seen in Table 1.

**Table 1:** Prevalence of *Toxoplasma gondii* and *Neospora caninum* in wild boars by host characteristics and geographical descriptors, Brazil, 2024.

Variables	nº of wild boars	*Toxoplasma gondii*		*Neospora caninum*	
		% of positives	CI	% of positives	CI
Age					
Young	30	23.3% (7/30)	11.8–40.9	30.0% (9/30)	16.7–47.9
Adult	97	28.9% (28/97)	20.8–38.6	37.1% (36/97)	28.2–47.0
Sex					
Female	61	29.5% (18/61)	19.6–41.9	45.9% (28/61)	34.0–58.3
Male	66	25.8% (17/66)	16.7–37.4	25.8% (17/66)	16.7–37.4
Pregnancy					
Pregnant	21	38.1% (8/21)	20.8–59.1	33.3% (7/21)	17.2–54.6
Nonpregnant	40	25.0% (10/40)	14.2–40.2	52.5% (21/40)	37.5–67.1
State					
São Paulo	109	29.4% (32/109)	21.6–38.5	33.9% (37/109)	25.7–43.2
Paraná	3	0% (0/3)	–	0% (0/3)	–
Rio Grande do Sul	15	20% (3/15)	7.1–45.2	53.3% (8/15)	30.1–75.2
Biome					
Atlantic rainforest	91	31.9% (29/91)	23.2–42.0	31.9% (29/91)	23.2–42.0
Pampa grasslands	15	20.0% (3/15)	7.1–45.2	53.3% (8/15)	30.1–75.2
Cerrado savanna	21	14.3% (3/21)	5.0–34.6	38.1% (8/21)	20.7–59.1
Mesoregion					
Araraquara	4	25.0% (1/4)	4.6–69.9	25.0% (1/4)	4.6–69.9
Bauru	6	0% (0/6)	–	66.7% (4/6)	30.0–90.3
Itapetininga	14	21.4% (3/14)	7.6–47.6	42.9% (6/14)	21.4–67.4
Ribeirão Preto	45	33.3% (15/45)	21.4–47.9	26.7% (12/45)	16.0–41.0
São José do Rio Preto	40	32.5% (13/40)	20.1–48.0	35% (14/40)	22.1–50.5
Sudoeste Paranaense	3	0% (0/3)	–	0% (0/3)	–
Noroeste Rio-Grandense	15	20.0% (3/15)	7.1–45.2	53.3% (8/15)	30.1–75.2
Cities					
Américo Brasiliense	1	0% (0/1)	–	0% (0/1)	–
Barretos	1	0% (0/1)	–	0% (0/1)	–
Bebedouro	3	33.3% (1/3)	6.2–79.2	33.3% (1/3)	6.1–79.2
Botucatu	6	0% (0/6)	–	66.7% (4/6)	30.0–90.3
Cajobi	4	25.0% (1/4)	4.6–69.9	50% (2/4)	15.0–85.0
Capão Bonito	14	21.4% (3/14)	7.6–47.6	42.9% (6/14)	21.4–67.4
Colina	5	0% (0/5)	–	60.0% (3/5)	23.1–88.2
Icém	4	0% (0/4)	–	0% (0/4)	–
Matão	3	33.3% (1/3)	6.2–79.2	33.3% (1/3)	6.1–79.2
Monte Alto	1	0% (0/1)	–	100% (1/1)	20.6–100
Monte Azul Paulista	22	40.9% (9/22)	23.3–61.3	31.8% (7/22)	16.4–52.7
Morro Agudo	11	27.3% (3/11)	9.8–56.6	0% (0/11)	–
Paraíso	31	38.7% (12/31)	23.7–56.2	35.5% (11/31)	21.1–53.0
São Simão	2	100% (2/2)	34.2–100	0% (0/2)	–
Tapabuã	1	0% (0/1)	–	100% (1/1)	20.6–100
Ipiranga	3	0% (0/3)	–	0% (0/3)	–
Santo Antônio das Missoes	15	20% (3/15)	7.0–45.2	53.3% (8/15)	30.1–75.2
Season of the year					
Spring	30	36.7% (11/30)	21.9–54.5	50.0% (15/30)	33.1–66.8
Summer	8	37.5% (3/8)	13.7–69.4	0% (0/8)	–
Autumn	50	24.0% (12/50)	14.3–37.4	42.0% (21/50)	29.4–55.8
Winter	39	23.1% (9/39)	12.7–38.3	23.1% (9/39)	12.6–38.3
IFAT	108	32.4 (35/108)	20.5–35.9	41.7 (45/108)	32.8–51.1
PCR (brain)	112	0 (0/112)	–	0 (0/112)	–
PCR (tissue homogenate)	18	11.1 (2/18)	3.1–32.8	–	–
TOTAL	127	27.6% (35/127)	20.5–35.9	35.4% (45/127)	27.6–44.1

We did not observe *T. gondii* or *N. caninum* cysts or pseudocysts in histopathology in hematoxylin-eosin-stained wild boars’ tissue sections. However, cysts suggestive of *Sarcocystis* spp. were observed in muscular tissues and these results are still under investigation (data not shown). The most common pathological alterations were the presence of inflammatory infiltrate, hemorrhage, degeneration, necrosis, and bacterial colonies. Other alterations observed were brain perivascular cuffing and neuronophagia, pulmonary emphysema, lymphoid depletion in the spleen, glomerular sclerosis, and the absence of intestinal villi.

*Toxoplasma gondii* antibodies were detected in 35/108 (32.4%, CI 95% 20.5–35.9%) wild boar while *N. caninum* antibodies were observed in 45/108 (41.7%, CI 95% 32.8–51.1%) animals of the same species. IFAT endpoint titers for *T. gondii* of 64 (8/35 boar, 22.8%), 128 (8/35 boar, 22.8%), 256 (6/35 boar, 17.1%), 512 (6/35 boar, 17.1%), and 1024 (6/35 boar, 17.1%) were observed. *Neospora caninum* endpoint titers were 50 (7/45 boar, 15.5%), 100 (9/45 boar, 20%), 200 (8/45 boar, 17.8%), 400 (6/45 boar, 13.3%), 600 (6/45 boar, 13.3%), 800 (5/45 boar, 11.1%), 3200 (3/45 boar, 6.7%), and 12,800 (1/45 boar, 2,2%).

All brain samples tested negative for *T. gondii* and *N. caninum*, and 2/18 (11.1%, CI 95% 3.1–32.8%) tissue homogenate samples tested positive for *T. gondii* on endpoint PCR. It was not possible to ascertain the *T. gondii* genotype of the endpoint PCR-positive tissue homogenate samples through PCR-RFLP due to the low target DNA concentration.

Variables with a p-value <0.20 in the Fisher exact test and <0.10 in the simple logistic regression are summarized in Table 2. There were no selected variables for the multiple logistic regression and no risk factors (sex, pregnancy status, age, state, mesoregion, cities, biome, or season of the year) for *T. gondii* infection in wild boars were associated. Of the four selected variables to evaluate *N. caninum* infection, only sex and season of the year remained significant (Table 3). There was a significant difference between the risk of positivity in spring and winter, and the seroprevalence of *N. caninum* in female wild boars was statistically significantly higher than in males. The non-parametric Wilcoxon test revealed no differences between wild boar weight and prevalence for *T. gondii* infection (positive boars: 51.6 ± 26.3, negative boars: 51.9 ± 27.7), or *N. caninum* infection (positive boars: 48.3 ± 27.8 kg, negative boars: 53.8 ± 26.8 kg).

**Table 2:** Fisher's exact test and simple logistic regression p-values of *Toxoplasma gondii* and *Neospora caninum* infections in wild boars from Brazil, 2024.

Variables	*Toxoplasma gondii*		*Neospora caninum*	
	Fisher's Exact Test	Simple Logistic Regression^1^	Fisher's Exact Test	Simple Logistic Regression^a^
Age	0.64	–	0.52	–
Sex	0.69	–	0.03	0.02*
Pregnancy	0.38	–	0.18	0.16
State	0.55	–	0.16	0.09*
Biome	0.26	–	0.26	–
Mesoregion	0.61	–	0.25	–
Cities	0.37	–	0.13	0.02*
Season of the year	0.49	–	0.01	0.00*

*Variables selected for the multiple logistic regression (p < 0.1).

a Simple logistic regression of variables with p < 0.2 in the Fisher's exact test.

**Table 3:** Multiple logistic regression analysis (p < 0.05) of *Neospora caninum* infection in wild boars from Brazil, 2024.

Variables		OR	95%	C.I.	Coefficient	S.E	Z-statistic	P-value
Season								
Autumm × Winter		2.3627	0.9136	6.1100	0.8598	0.4848	1.7736	0.0761
Spring × Winter		3.2156	1.1218	9.2173	1.168	0.5373	2.174	0.0297
Summer × Winter		0.0000	0.0000	>1.0E12	−12.0704	271.2347	−0.0445	0.9645
Sex (Male × Female)		0.4262	0.1957	0.9281	−0.8528	0.3971	−2.1479	0.0317

**Test**	**Statistic**	**D.F.**	**P-Value**					
Score	150.431	4	0.0046					
Likelihood ratio	180.911	4	0.0012					

Blood samples were collected from 73 hunting dogs: 27 females and 46 males; 63 from São Paulo and 10 from Paraná. *Toxoplasma gondii* antibodies were detected in 62/73 (85%, CI 95% 75–91%) hunting dogs, while *N. caninum* antibodies were observed in 31/73 (42%, CI 95% 32–54%) animals of the same species (Table 4). Variables with a p-value <0.20 in the Fisher exact test and <0.10 in the simple logistic regression are summarized in Table 5. None of the four selected variables remained significant for *T. gondii* infection when analyzed with other variables. For *N. caninum* infection, out of the two selected variables, only the mesoregion remained significant (Table 6).

**Table 4:** Seroprevalence of *Toxoplasma gondii* and *Neospora caninum* infection in hunting dogs by sex, geographical mesoregions, feeding habits, and wild boar meat consumption, Brazil, 2024.

Variables	nº of dogs	*Toxoplasma gondii*		*Neospora caninum*	
		% of positives	CI	% of positives	CI %
Sex					
Female	27	74.1 (20/27)	55.3–86.8	44.4 (12/27)	27.6–62.7
Male	46	91.3 (42/46)	79.7–96.6	41.3 (19/46)	28.3–55.7
Mesoregion					
Araçatuba	23	100 (23/23)	85.7–100	60.9 (14/23)	40.8–77.8
Araraquara	10	90.0 (9/10)	59.6–98.2	10.0 (1/10)	1.8–40.4
Ribeirão Preto	20	50.0 (10/20)	29.9–70.1	10.0 (2/20)	2.8–30.1
São José do Rio Preto	10	100 (10/10)	72.2–100	100 (10/10)	72.2–100
Sudoeste Paranaense	10	100 (10/10)	72.2–100	40.0 (4/10)	16.8–68.7
Feeding habits					
Commercial dry food	42	73.8 (31/42)	58.9–84.7	19.0 (8/42)	9.9–33.3
Human food scraps	31	100 (31/31)	89.0–100	74.2 (23/31)	56.8–86.3
Wild boar meat consumption					
No	26	92.3 (24/26)	75.9–97.9	30.8 (8/26)	16.5–50.0
Yes (cooked)	20	60.0 (12/20)	38.7–78.1	50.0 (10/20)	30.0–70.1
Yes (raw)	27	96.3 (26/27)	81.7–99.3	48.1 (13/27)	30.7–66.0
TOTAL	73	84.9 (62/73)	75.0–91.4	42.5 (31/73)	31.8–53.9

**Table 5:** Fisher's exact test and simple logistic regression p-values of *Neospora caninum* and *Toxoplasma gondii* infections in hunting dogs from Brazil, 2024.

Variables	*Toxoplasma gondii*		*Neospora caninum*	
	Fisher's Exact Test	Simple Logistic Regression	Fisher's Exact Test	Simple Logistic Regression
Sex	0.09	0.06*	0.81	–
Mesoregion	0.00	0.00*	0.00	0.00*
Feeding habits	0.00	0.97	0.00	0.00*
Wild boar meat consumption	0.00	0.00*	0.32	–

^1^Simple logistic regression of variables with p < 0.2 in the Fisher's exact test.

*Variables selected for the multiple logistic regression (p < 0.1).

**Table 6:** Multiple logistic regression analysis (p < 0.05) of *Neospora caninum* infection in hunting dogs from Brazil, 2024.

Variables			OR	95%	C.I.	Coefficient	S.E	Z-statistic	P-value
Mesoregion									
Araraquara × Araçatuba			0.0714	0.0077	0.6638	−2.6391	1.1374	−2.3203	0.0203
Ribeirão Preto × Araçatuba			0.0714	0.0133	0.3847	−2.6391	0.8591	−3.0718	0.0021
São Jose do Rio Preto × Araçatuba			987155.9889	0.0000	>1.0E12	13.8026	391.8647	0.0352	0.9719
Sudeste Paranaense × Araçatuba			0.428.6	0.0940	1.9540	−0.8473	0.7741	−1.0946	0.2737

**Test**	**Statistic**	**D.F.**		**P-Value**					
Score	29.70390	4		0,0000					
Likelihood ratio	35.78130	4		0,0000					

## 4 Discussion

Our findings revealed an overall *T. gondii* prevalence of 27.6% and a seroprevalence of 32.4% within the studied wild boar samples. In comparison, other studies have reported varying prevalence rates of *T. gondii* in free-ranging wild boars across different regions of Brazil. Santos et al. (2016) reported a lower *T. gondii* prevalence of 14.28% in Rio Grande do Sul state using bioassay. Similarly, Machado et al. (2019), Brandão et al. (2019), and Freitas et al. (2023) found a comparable seroprevalence of 21.1% in Paraná and Goiás states using the modified agglutination test, a molecular seroprevalence of 27% using the indirect hemagglutination test and endpoint PCR across Rio Grande do Sul, Santa Catarina, Mato Grosso, and São Paulo states, and a molecular prevalence of 26.58%, respectively. However, Machado et al. (2021) reported a higher seroprevalence of 76.9% in São Paulo state utilizing the IFAT method. A systematic review and meta-analysis demonstrated a 32% seroprevalence of *T. gondii* in North America, 26% in Europe, 13% in Asia, and 5% in South America (Rostami et al., 2017).

The seroprevalence of *N. caninum* found in this study was 41.7%, marking the first report of antibodies against *N. caninum* in wild boars from São Paulo state. In contrast, previous studies in Brazil found lower prevalence rates of *N. caninum* in free-ranging wild boars: Soares et al. (2016) reported a seroprevalence of 10.8% in Mato Grosso state using the IFAT method; Freitas et al. (2023) detected a molecular prevalence of 5.06% utilizing endpoint PCR in Rio Grande do Sul; and all 98 wild boar samples from Paraná and Goiás states were seronegative using the IFAT method (Kmetiuk et al., 2021). Globally, studies have reported varying prevalence rates of *N. caninum* in free-ranging wild boars. In the Czech Republic, Bártová et al. (2006) found a seroprevalence of 18.1% using inhibition ELISA. In Italy, Villa et al. (2023) reported a seroprevalence of 10.9% using the IFAT method, and Zanet et al. (2023) found a molecular prevalence of 37.5% in pregnant females and 25% in fetuses. In the USA, Cerqueira-Cézar et al. (2016) reported a seroprevalence of 15% using the agglutination test, and Bevins et al. (2013) reported a seroprevalence of 15.8% using competitive ELISA. In Spain, Almería et al. (2007) found a seroprevalence of 0.3% using IFAT. In Slovakia, Reiterová et al. (2016) reported a molecular prevalence of 20.4% using qPCR and seroprevalence of 33.6% using competitive ELISA. In Greece, Touloudi et al. (2015) found a seroprevalence of 1.1% using IFAT.

The observed differences in *T. gondii* and *N. caninum* prevalence across various studies could be attributed to biotic and abiotic factors influencing the parasites’ epidemiology, such as climate and the abundance of intermediate and definitive hosts. Even though there is no confirmation of their relationship in the epidemiological chain, wild carnivores such as maned wolves, cougars, and other small wild felids, ring-tailed coatis, crab-eating foxes, hoary foxes, striped hog-nosed skunks, as well as stray domestic cats and dogs, were observed directly or by footprints close to the study area and during hunting (Lux Hoppe, pers. obs.).

Furthermore, the lack of consistent and reliable test assays for detecting infections in wild boars could contribute to heterogeneity within and among studies (Rostami et al., 2017; Haydett et al., 2021). For instance, to determine the seroprevalence of *N. caninum* in wild boars, Haydett et al. (2021) employed two different commercially available ELISA test kits and IFAT. They noted discrepancies between duplicate sample results within and among test assays, with seroprevalence rates of the same samples ranging from 12.5% to 67.8% and 84.1%. Consistent with our findings, they also found no histopathological evidence of *N. caninum* in tissue samples (Haydett et al., 2021). A recent review highlighted the lack of comprehensive documentation regarding the histopathological diagnosis of *T. gondii* in pigs (Dubey et al., 2020). This method is deemed not sensitive due to the low density of tissue cysts in this species (Dubey, 2009a, Dubey, 2009b). The low tissue density non-homogeneous distribution of *T. gondii* could be related to the lack of concordance between the serological and molecular results in this study: The PCR could provide a false negative result if the tissue fragments obtained were insufficient (Truppel et al., 2010; Machado et al., 2021). A positive IFAT with negative PCR could also mean the animal was only exposed to the parasite in the past or that the host was in the chronic phase of infection and the parasites were absent in the analyzed samples (Truppel et al., 2010). Currently, the bioassay method is considered the ‘gold standard’ for demonstrating the presence of viable T. gondii (Opsteegh et al., 2020), and it is also used to increase the sensitivity of *N. caninum* molecular detection (De Barros et al., 2020), but due to ethical constraints, it could not be performed in this study.

Although our results showed no associated risk factor for *T. gondii* infection in wild boars, previous studies in various hosts reported an association between *T. gondii* seropositivity and age (Rostami et al., 2017). The significant associated risk factors for infection of *N. caninum* in wild boars were the higher seroprevalence in females and between spring and winter seasons.

Studies with free-ranging wild boars have reported similar *N. caninum* seroprevalence between males and females (Villa et al., 2023) or a higher seroprevalence in females, with a significant association (Bevins et al., 2013; Soares et al., 2016) or not (Cerqueira-Cézar et al., 2016; Reiterová et al., 2016). A higher *N. caninum* antibody prevalence in female wild boars may reflect different behaviors that impact exposure risk (Bevins et al., 2013). For cattle, it has been hypothesized that a recrudescence of infection and a subsequent rise of antibodies against *N. caninum* is more likely in females due to an immunosuppressive period during pregnancy (Wei et al., 2022), but it is worth noting that we found no statistical correlation between *N. caninum* seroprevalence and pregnancy. Further research is required to elucidate the mechanisms influencing the relationship between seropositivity for *N. caninum* and sex for wild boars.

In Brazil, there are no confirmed wild reservoirs of *N. caninum*, and dogs are considered the most likely reservoir species. To the authors' knowledge, no studies have investigated the elimination of *N. caninum* infection in swine. However, it appears that antibody levels against *T. gondii* decrease over time in this species (Opsteegh et al., 2011), and it is possible that a similar pattern could happen for both parasites since they are phylogenetically related. In the studied areas wild boars are more frequently hunted during the warmer seasons (spring and summer, Lux-Hoppe, pers. obs.), and the results may suggest they get infected during the warmer months, with antibody titers subsequently declining in the following months.

The hunting dog samples in this study had higher seroprevalence rates for both *T. gondii* (84.9%) and *N. caninum* (42.5%) compared to similar studies in Brazil using the same diagnostic test, IFAT. For instance, Machado et al. (2019) found a *T. gondii* seroprevalence of 31.8%, while Kmetiuk et al. (2021) reported an *N. caninum* seroprevalence of 9%. Both studies were conducted in the states of Paraná and Goiás. Additionally, Caramalac et al. (2021) observed seroprevalence rates of 35.9% for *T. gondii* and 15.4% for *N. caninum* in Mato Grosso do Sul. Furthermore, the seroprevalence of antibodies against both parasites in hunting dogs in this study exceeded rates reported in studies from other regions of the world: 19.6% for *T. gondii* using the latex agglutination test in Taiwan (Fan et al., 1998); 15% for *N. caninum* and 24% for *T. gondii* using IFAT in Spain (Machačová et al., 2016); 52.8% for *T. gondii* using the modified agglutination test in Spain and Africa (Cano-Terriza et al., 2016); and a mean of 36.8% for *T. gondii* using ELISA and modified agglutination test in Algeria (Bellatreche et al., 2022).

We obtained a multiple logistic model where the consumption of wild boar meat was identified as a significant risk factor exclusively for *T. gondii* among hunting dogs in this study. Nevertheless, caution is required when interpreting this result. If *T. gondii* is being transmitted through wild boar meat, it is likely to occur with the consumption of raw meat. Our results indicated that 96.3% of dogs fed raw wild boar meat tested positive for *T. gondii*, compared to 60.0% of dogs that consumed cooked wild boar meat. However, the prevalence among dogs that did not consume wild boar meat was 92.3%. When comparing these two groups (dogs that don't consume wild boar meat and dogs that consume cooked wild boar meat versus dogs that consume raw wild boar meat) the statistical differences disappear, suggesting that other factors not considered in this study may be involved. *Leptospira* serovars associated with synanthropic rodents were detected in the urine of dogs from the same kennels as this study (data not shown), suggesting that contact with intermediate hosts could be one of the unconsidered factors.

Due to their outdoor lifestyle and the practice of hunters providing raw game meat and offal to their dogs as rewards and dietary supplementation, hunting dogs were more susceptible to infections with both *T. gondii* and *N. caninum* in Spain (Machačová et al., 2016). A previous study reported hunting activity as one of the main risk factors associated with *T. gondii* infection in dogs (Cano-Terriza et al., 2016). Although the association between the consumption of wild boar meat and *N. caninum* seroprevalence in hunting dogs was not significant in this study, the ingestion of raw or undercooked meat from intermediate hosts is acknowledged as a risk factor for infection for both parasites (Dubey et al., 2017; Ducrocq et al., 2021).

Additionally, even when the consumption of human food scraps was not a significant risk factor for infection with *T. gondii* and *N. caninum* among hunting dogs, this practice can favor the transmission of both parasites due to an increased chance of the animals ingesting oocysts and viable tissue cysts (Silva et al., 2010; Dubey et al., 2017). Seroprevalence for *T. gondii* and *N. caninum* in hunting dogs was heterogeneous among and within the studied areas. The animals from which samples were obtained are usually taken long distances from their places of residence to hunt, so it was not possible to precisely ascertain their true living area. Infection by both parasites is multifactorial and associated with various risk factors that can vary between the studied areas, such as environmental contamination, the density of intermediate and definitive hosts, climate, diet, and water treatment, among others (Dubey, 2021; Mimoun et al., 2022). Sex was not a risk factor for *T. gondii* and *N. caninum* infection in hunting dogs, consistent with other studies that did not find sexual susceptibility to the infection in dogs (Gao and Wang, 2019; Gebremedhin et al., 2021).

Toxoplasma gondii, a zoonotic parasite, poses significant public health concerns, particularly for immunocompromised individuals and pregnant women (Almería and Dubey, 2021). It stands as one of the most fatal foodborne pathogens in the USA (Scallan et al., 2011), and ranks fourth among the most important parasites worldwide (FAO/WHO, 2014). Brazil has reported a T. gondii foodborne outbreak attributed to the consumption of undercooked pork meat (Darde et al., 2020). Two wild boar hunters from São Paulo state had neurotoxoplasmosis related to ingestion of wild boar meat. Both related the consumption of freshly hunted young females roasted in a barbecue while cutting and preparing the other hunted animals for freezing (Lux Hoppe, pers. comm.). The T. gondii genotype DB#6, highly pathogenic for mice and humans, was detected in wild boars from northern São Paulo State (Machado et al., 2021). Moreover, T. gondii inflicts substantial economic losses due to reproductive disorders in some production animals, posing risks to human health when these animals are destined for human consumption (Dubey, 2009a, Dubey, 2009b; Opsteegh et al., 2016). Additionally, recent studies suggest a higher prevalence of T. gondii infections in wildlife than previously recognized, affecting a diverse range of species and raising conservation concerns (Almería and Dubey, 2021). Understanding the roles of animal reservoirs in the spread of T. gondii infection is also crucial for controlling the dissemination of this parasite (Almería and Dubey, 2021). Wild boars may represent a key sentinel of environmental contamination with T. gondii and play an important role in maintaining its sylvatic cycle due to their scavenging and predatory behavior and frequent interactions with a wide range of different hosts (Sini et al., 2024).

Neospora caninum is recognized for inducing specific neuromuscular clinical signs in dogs (Dubey and Schares, 2011). It stands as one of the leading causes of abortions in cattle worldwide, resulting in annual losses exceeding a billion dollars for producers (Reichel et al., 2013). With a wide array of wild intermediate hosts identified (Zanet et al., 2023), recent research suggests that wild boars could serve as effective surveillance tools for this parasite (Haydett et al., 2021). Future investigations should prioritize confirming their role as both intermediate and potentially definitive hosts of N. caninum (Haydett et al., 2021).

Since 2013, when Brazilian environmental authorities permitted registered hunters to control wild boar populations, both the pursuit of this activity and the consumption of wild boar meat by hunters and their hunting dogs have increased gradually. By 2019, 44,258 registered hunters and 150 hunting enterprises were involved in this endeavor. However, by 2022, these numbers saw a significant surge, reaching 136,528 hunters and 361 companies registered (e-SIC, 2024). In São Paulo state, the second highest Brazilian state in registered hunters, 84% of them affirm consuming wild boar meat regularly (Machado et al., 2021). As wild boar meat becomes increasingly popular, it is important to recognize the potential health risks for people and animals who handle or consume it raw or undercooked. Proper management of offal and carcasses after hunts is crucial, and hunters should adhere to general hygienic procedures while processing the raw meat. Thorough cooking is essential before both human and animal consumption. (Almería et al., 2021). There is a need for heightened awareness of toxoplasmosis among hunters and healthcare providers. Without such awareness regarding the potential risk of exposure to game meat, patients may not promptly disclose relevant risk factors (Schumacher et al., 2021). The state of São Paulo has taken measures to monitor wild boar pathogens and manage carcass disposal to mitigate risks to human and animal health. Periodic educational initiatives are conducted for hunters, providing information on diseases, including zoonoses, associated with wild boars, along with guidance on sample collection and carcass disposal (SAA-SP, 2021). Given the potential risk of *T. gondii* transmission to hunters and their dogs, as well as *N. caninum* transmission to dogs, these pathogens should be highlighted in the course materials to aid in disease prevention for humans and hunting dogs.

## 5 Conclusion

The presence of antibodies against *T. gondii* and *N. caninum* in the studied wild boars and hunting dogs, as well as the detection of *T. gondii* DNA in the studied wild boars, suggests the circulation of these parasites in these animals. Educational actions directed towards hunters should highlight information on the prevention of these important foodborne diseases.

## Funding

This work was supported by the 10.13039/501100003593National Council for Scientific and Technological Development (10.13039/501100003593CNPq) [grant numbers 311063/2022–5 and 407,965/2021–1]; Higher Education Personnel (10.13039/501100002322CAPES) [grant number 001]; and the São Paulo Research Foundation (10.13039/501100001807FAPESP) [grant number 22022/13,548–3].

## 6 Declarations of interest

None.

## CRediT authorship contribution statement

**Patricia Parreira Perin:** Writing – review & editing, Writing – original draft, Visualization, Validation, Software, Resources, Project administration, Methodology, Investigation, Formal analysis, Data curation, Conceptualization. **Carmen Andrea Arias-Pacheco:** Software, Methodology, Investigation, Formal analysis. **Lívia de Oliveira Andrade:** Methodology, Investigation, Data curation. **Jonathan Silvestre Gomes:** Investigation, Data curation. **Adrian Felipe de Moraes Ferreira:** Investigation, Data curation. **Rafael Oliveira Pavaneli:** Investigation, Data curation. **Fabiana Alves Loureiro:** Investigation, Data curation. **Ana Luíza Franco:** Investigation, Data curation. **Wilson Junior Oliveira:** Data curation. **Talita Oliveira Mendonça:** Data curation. **Natália de Oliveira Zolla:** Data curation. **Mateus de Souza Ribeiro Mioni:** Methodology, Formal analysis. **Rosangela Zacarias Machado:** Resources, Methodology. **Luiz Daniel de Barros:** Resources, Methodology, Investigation, Formal analysis, Data curation. **João Luis Garcia:** Resources, Methodology, Investigation, Formal analysis, Data curation. **Rafaela Maria Boson Jurkevicz:** Investigation, Data curation. **Ana Carolina Cavallieri:** Investigation, Data curation. **Estevam G. Lux Hoppe:** Writing – review & editing, Writing – original draft, Visualization, Validation, Supervision, Software, Resources, Project administration, Methodology, Investigation, Funding acquisition, Formal analysis, Data curation, Conceptualization.

## Declaration of competing interest

The authors declare the following financial interests/personal relationships which may be considered as potential competing interests:Adrian Felipe De Moraes Ferreira reports financial support was provided by State of Sao Paulo Research Foundation. Jonathan Silvestre Gomes, Rafael Oliveira Pavanelia reports financial support was provided by Coordination of Higher Education Personnel Improvement. Estevam G. Lux Hoppea reports financial support was provided by 10.13039/501100003593National Council for Scientific and Technological Development. If there are other authors, they declare that they have no known competing financial interests or personal relationships that could have appeared to influence the work reported in this paper.
